# Cost-effectiveness of respiratory syncytial virus prevention strategies in Mozambique: a modelling study

**DOI:** 10.7189/001c.137870

**Published:** 2025-06-11

**Authors:** Esperança L Guimarães, Neele Rave, Assucênio Chissaque, Braiton Maculuve, Tufária Mussá, Cesar Palha, Farina L Shaaban, Louis J. Bont, Clint Pecenka, Nilsa de Deus, Andrew Clark, RSV GOLD III – Health Economics Study Group

**Affiliations:** 1 Instituto Nacional de Saúde, Marracuene district3943, Maputo, Mozambique; 2 Instituto de Higiene e Medicina Tropical, Universidade Nova de Lisboa, Lisbon, Portugal; 3 Department of Paediatrics, University Medical Centre Utrecht, Utrecht, The Netherlands; 4 Global Health and Tropical Medicine, Instituto de Higiene e Medicina Tropical, Universidade Nova de Lisboa, Rua da Junqueira 100, 1349-008 Lisboa, Portugal; 5 Ministério da Saúde, Maputo, Mozambique; 6 Universidade Eduardo Mondlane-Faculdade de Medicina, Maputo, Mozambique; 7 Center for Vaccine Innovation and Access, PATH, Seattle, WA, USA; 8 Department of Health Services Research and Policy, London School of Hygiene & Tropical Medicine, London, UK

**Keywords:** RSV, maternal vaccine, monoclonal antibody, cost-effectiveness analysis, low-income country

## Abstract

**Background:**

Respiratory syncytial virus (RSV) is a leading cause of severe acute lower respiratory infections in children. The World Health Organization recently recommended two passive RSV immunisation strategies for global use, but prices are yet to be determined in low- and middle-income countries (LMICs).

**Methods:**

We used a static cohort model to generate preliminary estimates of the potential health impact and cost-effectiveness of a maternal vaccine (RSVPreF, Abrysvo^®^, Pfizer) and a long-acting infant mAb (Nirsevimab, Beyfortus^®^, AstraZeneca, and Sanofi) over a 10-year period (2025–2034) in Mozambique. We incorporated cost-of-illness data from a recent study conducted in Maputo, Mozambique, and efficacy data from recent clinical trials. We compared each RSV prevention strategy to the current status quo (no pharmaceutical RSV prevention strategy). The primary outcome was the cost per disability-adjusted life year (DALY) averted from a government perspective, assuming year-round dose administration. We ran a range of deterministic scenarios, including a societal health perspective and a seasonal dose administration strategy. We also ran probabilistic uncertainty analyses and estimated the probability that each intervention would be cost-effective over a range of cost-effectiveness thresholds.

**Results:**

Year-round administration of a maternal vaccine (USD 5/dose, 69% efficacy, 87% coverage, 6 months protection) could cost USD 80 million and prevent 4,671 RSV deaths. Year-round administration of the long-acting infant mAb (USD 5/dose, 77% efficacy, 94% coverage, 5 months protection) could cost USD 85 million and prevent 5,128 RSV deaths. Over half the cost of the respective programs would be offset by healthcare cost savings. Compared to the current status quo, the cost per DALY averted from a government perspective was USD 288 (95% uncertainty interval 140–574) for the maternal vaccine and USD 289 (95% uncertainty interval 160–583) for the mAb. At an intervention price of USD 5 per dose, the probability is around 20% that either intervention is cost-effective at a threshold of 0.4 times the national GDP per capita.

**Conclusions:**

New passive immunisation interventions have the potential to prevent a substantial number of infant deaths in Mozambique. Both interventions have the potential to be cost-effective if priced below USD 5 per dose. A seasonal strategy could further improve cost-effectiveness if feasible to implement.

## INTRODUCTION

Respiratory Syncytial Virus (RSV) is a common viral pathogen that can lead to acute lower respiratory infections (ALRI) in young children.^1^ In 2019, an estimated 101,400 RSV-associated deaths occurred in children aged under five years of age worldwide. Over 95% of the RSV-ALRI cases and deaths occurred in low- and middle- income countries (LMICs).^2^ In Mozambique, respiratory diseases are responsible for around one in five deaths in children under 5 years old.^3^ Evidence on RSV in Mozambique is limited. However, in Maputo City, RSV has been detected in 23–27% of inpatients under 2 years of age with ALRI,^4^ and in 35% of patients of the same age with ALRI admitted to the intensive care unit (ICU).^5^

Forthcoming pharmaceutical interventions have the potential to significantly reduce the burden of RSV-ALRI. Currently, more than 20 vaccines and monoclonal antibody (mAb) candidates are at different stages of development.^6^ Recently, the World Health Organization (WHO) Strategic Advisory Group of Experts on Immunization recommended two interventions, a maternal vaccine (RSVPreF, Abrysvo^®^, Pfizer) and a long-acting infant mAb (Nirsevimab, Beyfortus^®^, AstraZeneca, and Sanofi) for global use. These interventions are starting to be introduced in the Americas and Europe.^7^ With maternal vaccination, RSV-neutralising antibodies are transferred across the placenta to the infant, offering protection to newborns.^8^ Phase III trials for the bivalent prefusion F protein-based maternal vaccine, RSVpreF, showed 82% efficacy against severe medically attended ALRI due to RSV after 3 months post birth, and 69% efficacy after 6 months post birth.^8^ In addition, the long-acting infant mAb, administered at birth in a single dose has shown 79% efficacy against medically attended RSV-associated ALRI after 5 months of follow-up.^9^

RSV interventions, including both the maternal vaccine and long-acting infant mAb, were considered by Gavi, the Vaccine Alliance, as one of six priorities of its vaccine investment strategy for the 2021–2025 funding period and beyond.^10^ One of the criteria Gavi considers is whether interventions offer good value for money. Mozambique is a low-income country where healthcare budget limitations and healthcare access inequalities are evident^11^ so it is particularly important to assess the health and economic impact of health spending decisions. There are currently few economic evaluations of RSV interventions in the African context. An assessment of the potential cost-effectiveness of these interventions in Mozambique is therefore needed to help inform decisions about whether they should be considered for inclusion in the national Expanded Program on Immunization (EPI), considering health priorities and budget limitations in the country.

This study aims to assess the potential health and economic impact of the recently prequalified maternal vaccine and long-acting infant mAb in Mozambique. This should provide important evidence to inform national policy decisions on the use of these interventions.

## METHODS

### STUDY DESIGN AND MODEL

In the base case scenario, we estimated the potential health impact and cost-effectiveness of a single dose of a maternal vaccine and a single dose of long-acting infant mAb from a government health perspective, including all costs borne by the public health system. We also ran a separate scenario analysis from a societal health perspective, which included additional direct medical and non-medical costs borne by households, as well as indirect costs. The health benefits were quantified using DALYs averted. We chose to use DALYs because they account for years lost due to premature mortality and years lived with the disability due to the disease, facilitating comparison with other potential public health interventions and results from other jurisdictions.^12^

We used the universal vaccine decision support model (UNIVAC version 1.7) to calculate the potential impact and cost-effectiveness of implementing each intervention over ten successive birth cohorts (2025–2034). UNIVAC is a proportionate outcomes static cohort model specifically designed for ease of use by country-led multidisciplinary teams in LMICs. It allows users to supplement default data with local information and provides a context-specific assessment of cost-effectiveness in settings with limited time, capacity, and resources. The model was developed in Microsoft Excel (Excel, Microsoft Corp, Redmond, Washington, United States). Detailed methods have been described elsewhere.^13^ Although the core methodology aligns with previous studies, we adjusted the input parameters to reflect the conditions in Mozambique.^14,15^

We compared both interventions to the current status quo (no RSV pharmaceutical prevention strategy) and to each other. In the base case scenario, we assumed both interventions were administered year-round, but also ran a separate scenario assuming seasonal administration. We analysed the new long-acting infant mAb, rather than Synagis^®^ (Palivizumab; short-acting mAb), because the new mAb is suitable for use in LMICs in all infants, not just those at high-risk, and is likely to have a lower price per course,^16^ while Palivizumab is a short-lasting mAb that requires up to five monthly doses to protect throughout the RSV season—making it an expensive and impractical prospect for most countries.^17^

We estimated the number of RSV severe and non-severe disease cases, outpatients, hospital admissions and deaths, with and without each RSV intervention, between birth and five years of age, using rates per 100,000 children aged < 5 years and assuming a range of possible pathways of the disease (Supplementary Figure 1). DALYs were calculated over the lifetimes of all ten birth cohorts evaluated. In addition, we calculated the program costs associated with each RSV prevention strategy and the healthcare costs averted.

The primary outcome measure was the cost per DALY averted, which reflected the lifetime costs and consequences calculated for all ten birth cohorts combined (2025–2034), from the government health perspective. We used 2023 USD for all costs. Future health outcomes and costs were discounted at 3% per year to reflect their present value in the first year of the RSV intervention program (2025).^18^ In addition to analysing cost-effectiveness from a government health perspective, we also evaluated the costs per DALY averted (in 2023 USD) from a societal health perspective, which included costs borne by households.

To interpret the cost-effectiveness ratios (USD per DALY averted) we compared them to a range of willingness-to-pay (WTP) thresholds (i.e., what the government might be prepared to pay for each DALY averted).^19^ Since Mozambique has not yet defined a country-specific WTP threshold, we assumed 0.4 times the national gross domestic product (GDP) per capita based on a study that estimated national cost-effectiveness thresholds using data on health spending and outcomes in Mozambique.^20^ This is equivalent to USD 249, considering the national GDP per capita of USD 608.4 in 2023.^21^ Further we also calculated the probability that the RSV interventions would be cost-effective over a range of thresholds (0.5 and 1.0 GDP per capita) expressed as proportions of the Mozambican GDP per capita.

To build consensus on the inputs used in the analysis, we invited 12 multidisciplinary experts to attend a one-day stakeholder consultation workshop in Maputo City, Mozambique on October 23, 2023. Participants included stakeholders from the EPI, the National Immunization Technical and Advisory Group (known in Mozambique as Comité de Peritos de Imunização), the Maternal and Child Health Program, the Medicine School of the Eduardo Mondlane University, PATH, Wilhelmina Children’s Hospital – UMC Utrecht (The Netherlands), WHO, and the United Nations Children’s Fund.

### BURDEN OF DISEASE

The disease burden inputs are presented in Table 1. For the rate of RSV-ALRI community cases (per 100,000 per year aged under five years of age) we used estimates for Mozambique as reported in a systematic review by Li et al.^2^ Using the same review, we assumed 10% of RSV-ALRI cases would be severe (require hospital admission) based on the proportion of RSV-ALRI cases with chest-wall indrawing from the low-income stratum. To estimate the rate of RSV-ALRI hospitalisation, we multiplied the rates of RSV-ALRI severe cases by the coverage of Bacillus Calmette-Guerin (BCG) vaccine (94%), assuming this would be a reasonable proxy for access to public hospitals for severe conditions^22^ and further assumed that each hospital admission would have had at least one clinic visit (either before or after admission). For the non-severe cases, we multiplied the rates of RSV-ALRI by the percentage (73%) of children under five years old with non-severe acute respiratory infection symptoms that received healthcare from the public sector, as reported in the 2023 Mozambique Demographic and Health Survey (DHS).^11^ We multiplied the number of severe RSV-ALRI hospitalisations by the in-hospital fatality ratio reported for children under five years old in a systematic review study that included 19 LMICs (1.5%), including Mozambique.^23^ Consistent with an RSV mortality study from Zambia, we assumed that around two-thirds of RSV deaths before five years of age would occur in the community rather than in hospitals. This assumption, combined with other burden parameters gave a fatality ratio of around one-third for severe cases that did not have hospital access. We cross-checked our estimates of the number of RSV deaths (around 1,200 each year), with those derived by assuming that 2% of all under-five deaths would be attributed to RSV. This proportion, estimated in Child Health and Mortality Prevention Surveillance (CHAMPS)^23^ and Li et al.,^2^ resulted in slightly higher estimates of RSV deaths (around 1,600 each year), and suggested our base case estimates were slightly conservative. Different burden input sources are shown in Supplementary Figure 2. We conservatively assumed that RSV mortality rates would decline each year in the absence of pharmaceutical RSV interventions, and assumed this trend would be consistent with the overall trend in projected under-five mortality rates from the United Nations World Population Prospects.^24^

For the age distribution of RSV disease, we used data from a cost-of-illness (COI) study conducted in Maputo during the 2023 RSV season^28^ and fit parametric Burr curves to these data to generate estimates by week of age under five years. This study included all children under two years of age with ALRI that visited an outpatient clinic or were admitted to the Maputo Central Hospital. The age distribution for RSV outpatient cases was used for non-severe RSV cases and clinic visits, while the age distribution for RSV inpatient cases (excluding ICU cases) was used for severe RSV cases, clinic visits and hospital admissions. We used the age distribution among RSV cases admitted to the ICU as a proxy for the age distribution of RSV deaths. We estimated the proportion of RSV counts expected to occur in the missing age range (24–59 months) based on preliminary estimates from a review of datasets in LMICs.^34^ We compared the age distributions obtained from the COI study with age distributions reported in a study by Pale and colleagues in 2017 and 2018^4^ (see Supplementary Figure 3).

### RSV INTERVENTION IMPACT

We estimated the health impact of each intervention for each week of age based on the expected RSV burden, intervention coverage, and efficacy assumed for each week of age (Table 1). For the maternal vaccine, coverage for year-round administration was assumed to be 87%, based on at least one antenatal care (ANC) attendance, as recorded in Mozambique during the 2022 Demographic and Health Survey (DHS). The national tetanus vaccine coverage estimate of 48% was used as the lower bound,^11^ while the upper bound was assumed to be at 95%. For simplicity, we did not adjust for gestational age and assumed efficacy would be consistent with the results from the recent clinical trial.

For year-round administration of the long-acting infant mAb we assumed coverage would be equivalent to the mean coverage of BCG vaccine (94%) in Mozambique from 2015 to 2019, as reported by WHO/UNICEF estimates of national immunisation coverage (WUENIC).^29^ We used coverage data from this period because the COVID-19 pandemic negatively impacted health services delivery, including immunisation services^35^; in Mozambique, BCG vaccine coverage saw an average decrease of 10% during the pandemic. We assumed an uncertainty range from 79% (the current coverage estimate)^29^ and 98%. We also assumed real-world delays in the administration of the long-acting infant mAb, consistent with the timeliness of BCG as reported in the DHS 2011^36^ (i.e., the cumulative coverage at each week of age after birth).

We based efficacy estimates for each RSV intervention on evidence obtained from Phase III clinical trials. For the maternal vaccine, an efficacy of 69.4% was observed in preventing severe ALRI associated with RSV during the infantś first six months of life.^8^ In contrast, the long-acting infant mAb demonstrated an efficacy of 78.6% against very severe disease during five months following immunisation.^9^ For non-severe presentations, we assumed the efficacy to be 51.3% (97.58% CI: 29.4–66.8) for the maternal vaccine and 76.4% (95% CI: 62.3–85.2) for the long-acting infant mAb based on the reported efficacy against medically attended RSV.

### RSV INTERVENTION PROGRAM COSTS

The immunisation program cost comprises the vaccine price per dose, wastage, international transportation and handling, and incremental health system costs. Mozambique is currently in the initial self-financing Gavi phase, which normally allows the payment of USD 0.20 per dose of vaccine; however, the dose price and level of Gavi support for RSV interventions is currently uncertain. We therefore assumed a base case price of USD 5 per dose and considered two additional dose price scenarios of USD 2.5 and 15. We assumed these prices for both interventions^14^ and assumed they would remain fixed for the duration of the analysis.

### HEALTHCARE COSTS ASSOCIATED WITH THE TREATMENT OF RSV DISEASE

In the base case scenario, we used cost data obtained from a recent COI study on RSV in Maputo City. This study took place during one respiratory season from February 2023 to August 2023.^28^ Costs borne by the government included general operational costs per illness episode and direct medical costs (e.g., medication and imaging). Costs borne by households included any out-of-pocket direct medical costs not covered by the government as well as direct non-medical costs (e.g., transportation) and indirect costs (e.g., income lost) that occurred during the initial visit to a governmental facility or prior/post initial visit at any healthcare facility. Since the costs were all collected at a tertiary-level hospital, we adjusted them to account for the distribution of primary, secondary, and tertiary facilities across the country, and used cost estimates from WHO-CHOICE for Mozambique to inform the relative cost of care at primary, secondary, and tertiary hospitals. From the governmental perspective the study estimated USD 6.98 (IQR: 6.97–6.99) per outpatient clinic visit and USD 465.30 (IQR: 411.05–519.55) per RSV hospital admission. From the societal perspective, the study estimated USD 41.79 (IQR: 10.91–72.66) per RSV outpatient case and USD 523.01 (IQR: 466.07–579.94) per RSV hospital admission.^37^ We assumed the same outpatient cost per visit for severe and non-severe RSV cases (Table 1).

### UNCERTAINTY ANALYSIS

We ran a number of deterministic scenarios to understand how variations in the input parameters and assumptions might influence the cost-effectiveness results.^12^ The scenarios were defined based on parameters reported to be influential in previous studies^14,38,39^:

- Dose price of USD 2.5
- Dose price of USD 15
- Dose price of USD 25
- Excluding indirect costs
- Lower vaccine coverage
- High efficacy (upper bound of 95% CI)
- Low efficacy (lower bound of 95% CI)
- High healthcare costs (75^th^ percentile)
- Low healthcare costs (25^th^ percentile)
- Higher initial efficacy but reduced duration of protection for maternal vaccine (assuming efficacy of 81.8% and 57.1% for severe and non-severe RSV disease, respectively, for a fixed 3-month period).
- Gradual waning protection for both interventions (assuming a gamma function described elsewhere to assume efficacy is initially very high, then gradually approaches zero by age 12 months).^14^
- Seasonal dose administration

For scenario xi regarding seasonal dose administration specifically, we took into account the seasonal burden of RSV disease in the southern part of Mozambique, wherein much of the burden occurs between February and July.^4,28^ We evaluated various scenarios targeting specific months of the year (rather than year-round strategies) as well as various potential months of birth that could be targeted for protection using either intervention. Each combination of the expected health impact (percent reduction in RSV hospital admissions) and dose efficiency (doses required to achieve a 1% reduction in RSV hospital admissions) was compared to a year-round preventive strategy. Estimates of health impact by month of birth were calculated using a database of RSV hospital admissions, using the interval between birth and age of admission to determine the protection that could have been provided by either intervention, assuming different months of birth would qualify for protection. This analysis assumes protection from birth and no catch-up in dose administration at older ages. The database included data from a recent COI Study in Maputo, Mozambique, where during one respiratory season (from February to August 2022) all hospitalised children under two years of age were tested for RSV.^19^ In total, 109 RSV positive hospital admissions were recorded. Since children in the described study were only tested during a certain period of the year, we also repeated the analysis using data from another study by Pale and colleagues that was conducted in Maputo from 2017 to 2018.^4^

In addition to the aforementioned scenarios, we also ran a probabilistic sensitivity analysis (PSA) simultaneously varying all parameters within their ranges, using 1,000 Monte Carlo simulations. For both intervention options we ran a separate PSA for the base case (USD 5 per dose) and two alternative fixed price scenarios (USD 2.5 and USD 15 per dose). For simplicity, we assumed a transparent Beta-PERT distribution for all parameters and their respective ranges. The probabilistic results were presented on a cost-effectiveness plane. To estimate the probability that the intervention would be cost-effective at different WTP thresholds, the results were presented using cost-effectiveness acceptability curves. Finally, we estimated the maximum price per dose at which each intervention would be considered cost-effective at various thresholds, based on the base-case assumptions.

## RESULTS

All the base-case results are presented in Table 2. Without any pharmaceutical RSV intervention in Mozambique, we estimated 3.6 million RSV disease cases, 339,343 hospital admissions, and 10,816 deaths in ten birth cohorts of children (2025–2034). The long-acting infant mAb was estimated to have slightly more health benefit than the maternal vaccine, primarily due to the higher intervention coverage assumed for the mAb (Table 2). Either intervention was estimated to prevent more than 30% of the severe RSV disease cases and hospital admissions and more than 40% of RSV deaths in children under five years of age. Either intervention could prevent more than 4,500 RSV deaths over a 10-year period.

The maternal vaccine program could cost around USD 80 million over ten years, but this would be offset by USD 46 million healthcare costs averted from a government perspective (or USD 63 million from a societal perspective) (Table 2). The long-acting infant mAb could cost around USD 85 million over ten years, but this would be offset by USD 48 million healthcare costs averted from a government perspective (or USD 67 million from a societal perspective) (Table 2).

Both interventions had a very similar cost per DALY averted (from a government perspective) when compared separately to no intervention. The cost per DALY averted was USD 288 (95% uncertainty interval 140–574) for the maternal vaccine and USD 289 (95% uncertainty interval 160–583) for the long-acting infant mAb, corresponding to around 48% of the national GDP per capita (Table 2).

The cost per DALY averted was sensitive to changes in the dose price and efficacy assumptions. All scenarios generated a ratio below one times the national GDP per capita with the exception of four: the lower bound estimate of efficacy, high maternal vaccine efficacy for only 3 months, a dose price of USD 15, and a dose price of USD 25 (Supplementary Figure 4 and 5). A nine-month strategy targeting births from September to April could generate the same impact as a year-round strategy and require 25% fewer doses (Supplementary Figure 6).

**Figure 1.**
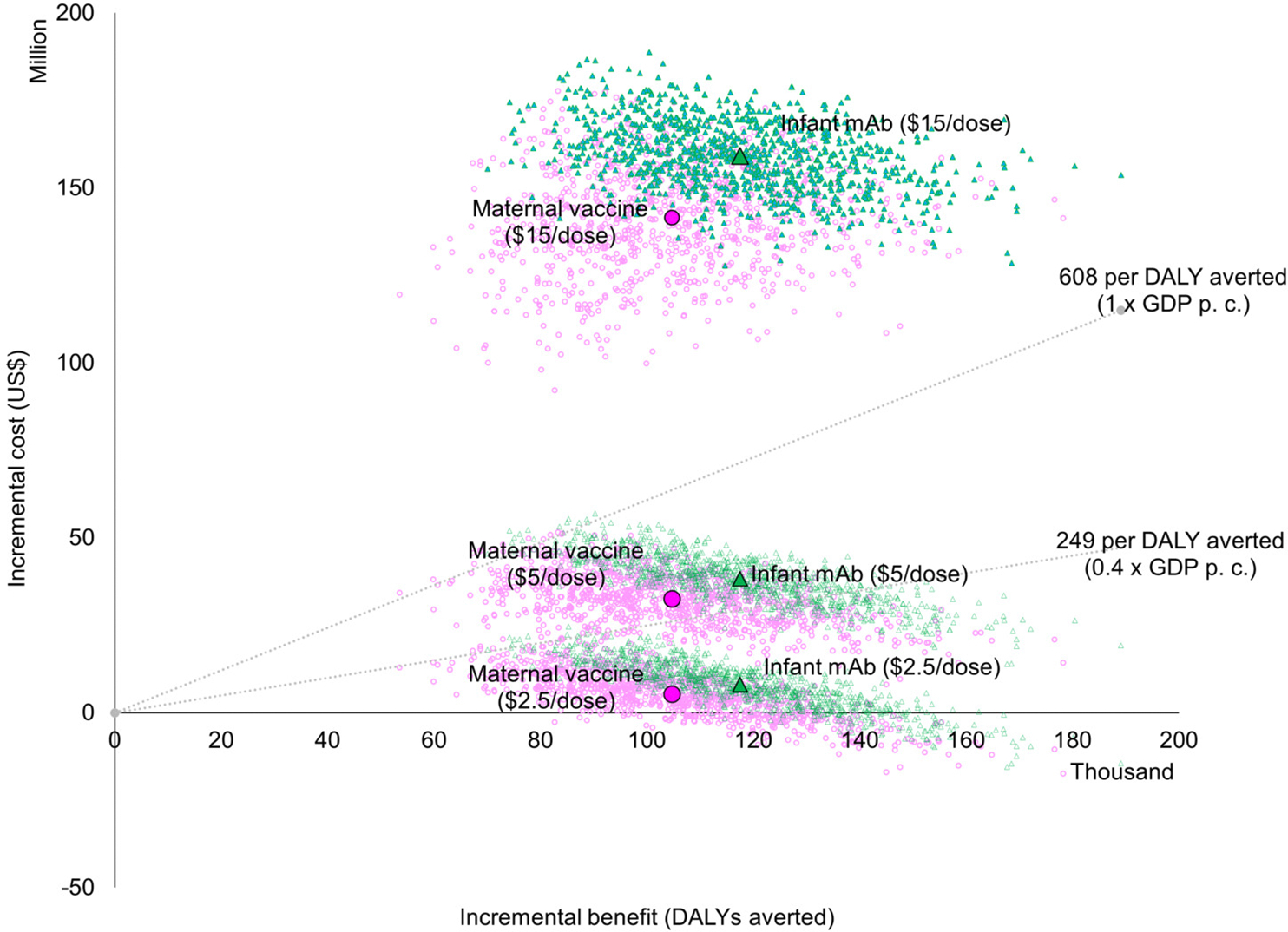
Probabilistic clouds of incremental costs and health benefits of both respiratory syncytial virus prevention strategies (maternal and long-acting infant monoclonal antibody immunization) assuming dose price at USD 2.5, USD 5, and USD 15 from a government perspective.

**Figure 2.**
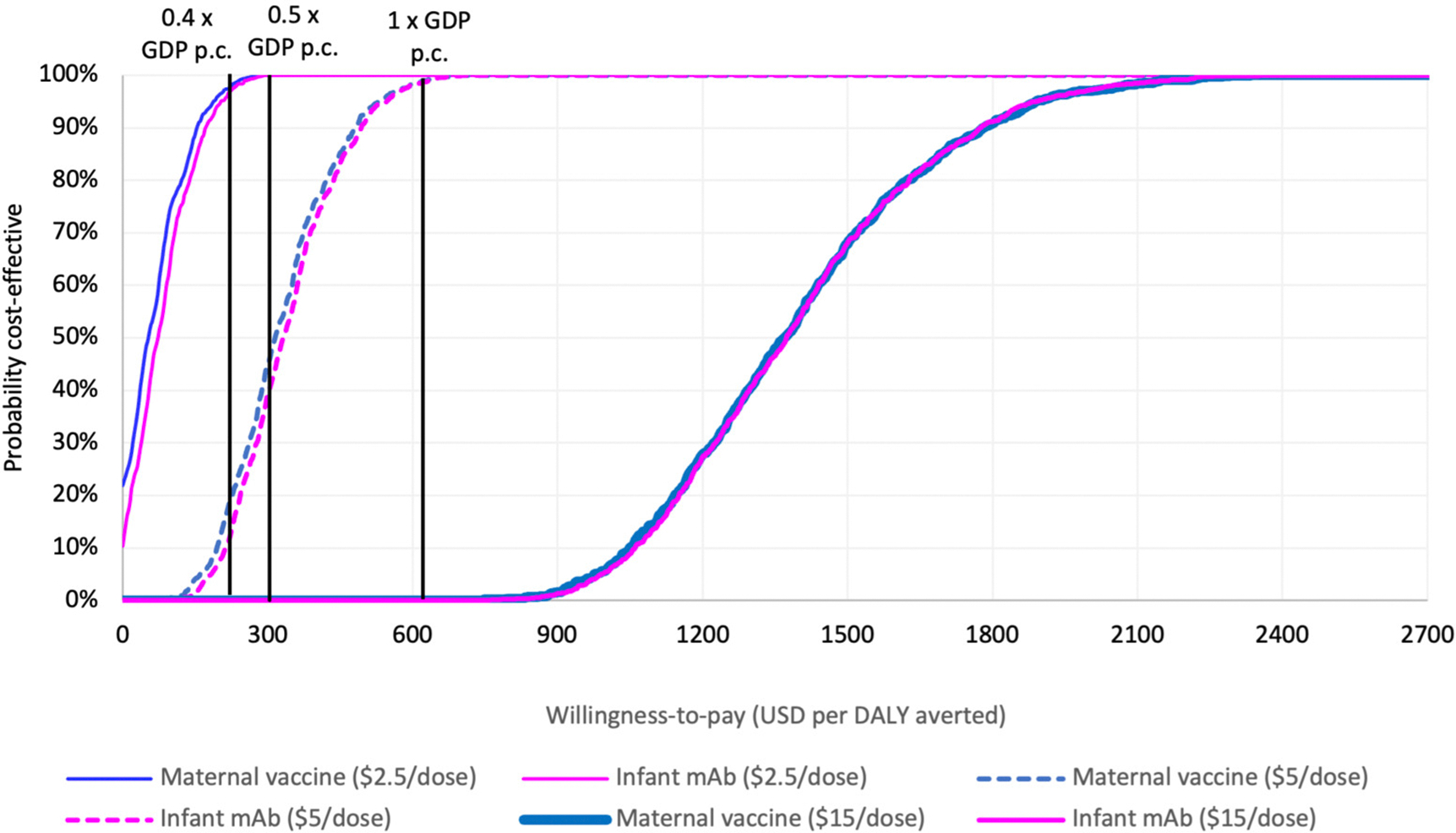
Probability that each respiratory syncytial virus prevention strategy (either maternal vaccine or long-acting infant monoclonal antibody) will be cost-effective compared to no intervention, at different willingness-to-pay thresholds.

Both interventions were estimated to generate similar value for money compared to the status quo when assuming the same price per dose, with overlapping probabilistic clouds of uncertainty for costs and DALYs averted (Figure 1). At an intervention price of USD 5 per dose, the probability is approximately 20% that either intervention would be cost effective at a threshold of 0.4 times the national GDP per capita. This probability increases to 50% at 0.5 times the national GDP per capita and reaches 100% at a threshold of 1.0 times the national GDP per capita. If the dose price reduces to USD 2.5, the probability of being cost-effective at the 0.4 times the national GDP per capita threshold increases to almost 100%. If it rises to USD 15, however, the probability that either intervention could be cost-effective decreases to zero percent (Figure 2).

## DISCUSSION

Both interventions included in our analysis were recently prequalified and recommended for global use by WHO and have started to be introduced in Argentina, Chile, Canada, the United Kingdom, and the United States.^40^ Our estimates indicate that both interventions have the potential to reduce the RSV burden in Mozambique, potentially averting over 4,500 deaths (a reduction of more than 40%) over a 10-year period. The impact was lower for non-severe RSV cases because a substantial proportion of these cases were estimated to occur in the six- to 59-month age range. A strength of our analysis is that we used model input parameters that were approved by local stakeholders, including estimates from Mozambique. In particular, we were able to incorporate the results of a recent cost-of-illness study conducted in Maputo.

While the market price per dose for LMICs has yet to be defined, the prices for the maternal vaccine and mAb are unlikely to be the same. mAb costs are likely to be higher than those of the maternal vaccine due to existing pricing agreements for vaccines in LMICs and lower production costs. Using the current study’s price estimation, both interventions were estimated to have a reasonable probability of being cost-effective at a per-dose cost of USD 5 and a WTP threshold of 0.4 times the national GDP per capita; however, the cost-effectiveness was more favourable when the dose price was reduced to USD 2.5. These results are conservative (from a government health perspective) because they assume the government will cover the full cost of the vaccine, and do not account for anticipated support from Gavi.

We did not evaluate a combined strategy (e.g., using long-acting infant mAb for newborns that were not previously protected by maternal vaccination), but this could be an effective way to increase protection of the birth cohort by providing two opportunities to provide protection. In Mozambique, a high number of zero dose children and women who have not had ANC visits remains.^41^ Addressing these gaps may require further analyses of how disease risk and access to care varies in different groups of the population.

Our results are comparable with previously published costs-effectiveness analyses in Vietnam and sub-Saharan African countries.^14,38,39^ In our analysis, the long-acting infant mAb was estimated to have a higher health impact, which was primarily due to the higher coverage assumed compared to the maternal vaccine. Other studies reveal a similar pattern.^14^ We considered a range of acceptable thresholds to interpret the results. In Vietnam, Do et al. assessed both preventive strategies and demonstrated that they have the potential to be cost-effective at USD 5 per vaccine dose, with a WTP threshold of 0.5 times the national GDP per capita.^15^ Koltai et al. conducted a study on the cost-effectiveness of both interventions in Kenya and South Africa, and found that the interventions were cost-saving in South Africa and cost-effective in Kenya at a price of USD 5.^39^ Baral et al. performed a similar study across several LICs, including in sub-Saharan Africa, which found that the cost per DALY averted was USD 949 for maternal vaccine and USD 257 for the long-acting infant mAb, based on a dose price of USD 3. This study, however, was based on earlier information about the clinical efficacy of the maternal vaccine. More recent estimates show equivalent cost-effectiveness for each option at equivalent prices.^38^ Other infant immunisation programs in Mozambique, such as for rotavirus vaccine, have recently been shown to be cost-effective in young children (USD 70 per DALY averted).^42^ The introduction of an RSV preventive intervention in Mozambique would also mitigate any future RSV outbreaks that might be associated with climate change i.e. higher temperatures and increased rainfall,^43^ as reported in the Americas^44,45^ and Madagascar (Africa).^45^

Our study had some limitations. First, we used a static cohort model that did not account for transmission dynamics. However, there is currently limited evidence on the extent to which either intervention will interrupt transmission. Second, we used data from the south of the country (Maputo) to estimate the age profile of RSV outpatients and inpatients, costs of RSV illness and seasonality. While we have no a priori reason to assume the age profile or seasonality would be different in the north and central regions, it is possible that the costs of illness could vary in different regions. However, deterministic scenarios with lower and higher healthcare costs did not alter the cost-effectiveness conclusions. It is also important to note that several other influential inputs and adjustments (e.g. RSV disease burden rates, DHS access to care, BCG and ANC coverage) were taken from national estimates, so we believe the analysis is as representative as it is possible to be at the present time. Third, clinical trials for both interventions have predominantly been conducted in high-income countries with lower rates of severe RSV outcomes compared to LMICs, such as Mozambique. Participants in controlled clinical trials often differ significantly from the average populations in LMICs, which may limit the generalisability of the findings. Fourth, we did not explore the potential balance of benefits to risks. In the Phase III trial, a numerical increase in preterm births was observed in some groups that received the maternal vaccine.^46^ In response to this safety signal, an ongoing randomised controlled study is investigating the effectiveness and safety of the maternal vaccine in several African countries.

We investigated seasonal administration of either the maternal vaccine or long-acting infant mAb to assess the impact and cost-effectiveness of a seasonal program rather than a year-round program. Our analysis shows that a nine-month seasonal administration strategy could give the same overall impact, but better dose efficiency, as compared to a year-round strategy. Introducing a seasonal approach could therefore optimise resource allocation; however, no seasonal administration of any vaccine currently occurs in Mozambique and the feasibility of this approach is unclear. This seasonal approach also assumes that the dose price negotiated for a year-round program would the same as the price negotiated for a seasonal program. Seasonal administration implementation poses several challenges because it would require healthcare personnel training as well as investment in education, logistics, and monitoring systems. These factors imply a substantial increase in system and delivery costs per dose. South Africa has adopted seasonal approaches for influenza,^47^ and several countries in Europe and America are following similar approaches for RSV vaccine administration.^48,49^ The experience of these countries could provide valuable insights, since they registered positive results on reducing burden of both diseases especially in high risk people.^50,51^ Further investigation in other African countries using similar strategies could also enhance the understanding of this approach in similar contexts.

The evidence generated is aligned with the current priority of the National Research Agenda 2024 – 2028,^52^ which supports the introduction of impactful and cost-effective interventions. Passive RSV immunisation has the potential to prevent a substantial number of RSV cases in infants, and to be cost-effective at prices below $5/dose.

Although both interventions have undoubted potential to reduce disease burden and be cost-effective in Mozambique, the decision to introduce them would depend on several other criteria including acceptability, safety, government health priorities, and budget availability. Our existing model and evaluation should provide a convenient basis for updating estimates of cost-effectiveness as updated national data emerges, particularly on the price of each intervention.

## CONCLUSION

A maternal vaccine or long-acting infant mAb could prevent a substantial number of infant deaths in Mozambique. Both interventions have the potential to be cost-effective in Mozambique if appropriately priced. Policymakers should consider introducing these interventions as part of the national immunization strategy and explore the potential for seasonal administration to maximize efficiency.

## Supplementary Material

Supplementary Document

SUPPLEMENTARY MATERIALS

Supplementary Materials

Download: https://joghep.scholasticahq.com/article/137870-cost-effectiveness-of-respiratory-syncytial-virus-prevention-strategies-in-mozambique-a-modelling-study/attachment/288339.pdf

## Figures and Tables

**Table 1.** Input parameters for estimating the impact and cost-effectiveness of maternal and long-acting infant mAb for RSV disease prevention in Mozambique

Input parameters	Central value	Uncertainty range	Sources

**Incidence (per 100,000 children under 5 years old per year) ^a^**			
Non severe RSV cases	5,247	3,870–7,110	^2^
Non severe RSV visits	3,841	2,941–5,404	^11^
Severe RSV cases	583	430–790	^2^
Severe RSV visits	583	430–790	^2^
Severe RSV hospitalizations	542	400–735	^22^
RSV deaths	23	12–31	Assumed based on^2,23,25^

**Disability weights (%)**			
Non-severe RSV	5.1	3.2–7.4	^26^
Severe	13.3	8.8–19	^26^

**Mean duration of illness (days)**			
Non-severe RSV	5	3–7	^27^
Severe	10	7–14	^27^

**Cumulative age-specific burden (non-severe)**			^28^
<1 month	4%	NA	
<2 months	4%		
<3 months	12%		
<6 months	24%		
<1 year	44%		
<2 years	71%		
<3 years	87%		
<4 years	95%		
<5 years	100%		
**Cumulative age-specific burden (severe)**			^28^
<1 month	7%	NA	
<2 months	10%		
<3 months	29%		
<6 months	53%		
<1 year	77%		
<2 years	92%		
<3 years	97%		
<4 years	99%		
<5 years	100%		
**Cumulative age-specific burden (death)**			^28^
<1 month	2%		
<2 months	13%		
<3 months	39%		
<6 months	72%		
<1 year	89%		
<2 years	96%		
<3 years	98%		
<4 years	99%		
<5 years	100%		

**Maternal immunization impact**			
Expected coverage (ANC proxy) (%)	87	48.0–95.7	Assumed based on^11^
Efficacy against RSV severe cases (%)	69.4	44.3–84.1	^8^
Efficacy against RSV non-severe cases (%)	51.3	29.4–66.8	^8^
Duration of protection (months)	6	3–7	^8^
**Infant mAb immunization impact**			
Expected coverage (BCG proxy) (%)	94	79–98	^29^
Efficacy against RSV severe cases (%)	78.6	48.8–91.0	^9^
Efficacy against non-severe RSV (%)	76.4	62.3–85.2	^9^
Duration of protection (months)	5	4–6	^9^

**Costs of intervention**			
Price per dose (USD)	5	NA	Assumption
Syringe price per dose	0.000	NA	Assumed pre-filled syringe
Safety box price per dose	0.012	NA	^30^
International handling (% price per dose)	3%	NA	^31^
International delivery (% price per dose)	6%	2%–15%	^32^
Incremental system cost per dose (USD)	2.21	1.72–2.93	^33^

**Wastage assumptions (% per dose)**			
Dose and pre-filled syringe	5%	NA	Assumption
Safety boxes/bags	5%	NA	Assumption
**Costs of RSV related care Governmental Perspective**			
**Non-severe disease (USD)**			
Outpatient visit cost	6.98	6.97–6.99	^28^
**Severe disease (USD)**			
Outpatient visit cost	6.98	6.97–6.99	^28^
Hospitalization cost	465.30	411.05–519.55	^28^

**Costs of RSV related care Societal Perspective**			
**Non-severe disease (USD)**			
Outpatient visit cost	41.79	10.91–72.66	^28^
**Severe disease (USD)**			
Outpatient visit cost	41.79	10.91–72.66	^28^
Hospitalization cost	523.01	466.07–579.94	^28^

All future costs and benefits are discounted at a 3% discount rate per year. Costs and GDP percentage are expressed in 2023 USD.

Abbreviations: ANC, antenatal care; BCG, Bacillus Calmette-Guerin; GDP, gross domestic product; mAb, monoclonal antibody; RSV, respiratory syncytial virus.

**Table 2.** Estimated health impact and costs of RSV preventive interventions (MI: USD 5/dose 87% coverage, 69.4% efficacy, 6 months protection; and mAb immunization: USD 5/dose, 94% coverage, 78.6% efficacy, 5 months protection) compared to no pharmaceutical intervention in Mozambique (2025–2034).

Outcomes	No intervention	Maternal vaccine	Long-acting infant mAb

**Lifetime costs and effects**			
RSV cases	3,650,126	3,186,112	3,067,442
RSV severe cases	365,013	247,337	244,023
RSV visits	2,769,838	2,398,630	2,310,871
RSV hospitalizations	339,343	229,943	226,861
RSV deaths	10,816	6,145	5,688
DALYs (discounted* in USD)	268,936	152,931	141,555
Vaccine program costs (discounted* in USD)	0	79,608,014	84,813,864
Government healthcare costs (discounted* in USD)	151,113,622	104,940,864	103,175,597
Societal healthcare costs (discounted* in USD)	248,823,930	186,048,717	181,494,226

**Differences (comparator = no vaccine)**			
RSV total cases	-	464,014	582,684
RSV severe cases	-	117,675	120,990
RSV visits	-	371,208	458,967
RSV hospitalizations	-	109,400	112,481
RSV deaths	-	4,671	5,128
DALYs (discounted*)	-	116,004	127,380
Vaccine program costs (discounted* in USD)	-	79,608,014	84,813,864
Government healthcare costs (discounted* in USD)	-	-46,172,757	-47,938,024
Societal healthcare costs (discounted* in USD)	-	-62,775,214	-67,329,704

**Cost (USD) per DALY averted (comparator = no vaccine)**			
*Government cost perspective*			
Cost (discounted* in USD)	-	33,435,257	36,875,840
DALYs averted (discounted* in USD)	-	116,004	127,380
Cost per DALY averted (discounted* in USD)	-	288	289
*Societal cost perspective*			
Cost (discounted* in USD)	-	16,832,800	17,484,160
DALYs averted (discounted* in USD)	-	116,004	127,380
Cost per DALY averted (discounted* in USD)	-	145	137

* All future costs and benefits are discounted at a 3% discount rate per year. Costs are in 2023 USD.

Abbreviations: DALY, disability-adjusted life year; GDP, gross domestic product; mAb, monoclonal antibody; MI, maternal immunization; RSV, respiratory syncytial virus.

## RSV GOLD III – Health Economics Study Group members, Mozambique COI (in alphabetical order of their surnames):

Louis Bont (Department of Paediatrics, University Medical Centre Utrecht, Utrecht, The Netherlands), Elias Manjate (Faculty of Medicine, University Eduardo Mondlane, Maputo), Yara Manjate (Faculty of Medicine, University Eduardo Mondlane, Maputo), Izilda Matimbe (Faculty of Medicine, University Eduardo Mondlane, Maputo), Tufária Mussá (Faculty of Medicine, University Eduardo Mondlane, Maputo), An Nguyen (Center for Vaccine Innovation and Access, PATH, Ho Chi Minh City, Vietnam), Clint Pecenka (Center for Vaccine Innovation and Access, PATH, Seattle, WA, USA), Neele Rave (Department of Paediatrics, University Medical Centre Utrecht, Utrecht, The Netherlands), Farina Leonie Shaaban (Department of Paediatrics, University Medical Centre Utrecht, Utrecht, The Netherlands), Cristina Sinussene (Faculty of Medicine, University Eduardo Mondlane, Maputo), Farida Zavala (Faculty of Medicine, University Eduardo Mondlane, Maputo). A complete list of names and affiliations of the multi-site group members can be found in the Online Supplementary Document.
