# Supplementary material for: Cost-effectiveness of respiratory syncytial virus prevention strategies in Mozambique: a modelling study
Source: J Glob Health Econ Policy. Author manuscript; Available in PMC 2025 Oct 7. (PMC12499514; doi:10.7189/001c.137870)
Supplement: Supplementary Document [file NIHMS2110951-supplement-Supplementary_Document.pdf]

Supplement to: Guimarães EL, Rave N, Chissaque A, et al.; RSV GOLD III – Health Economics Study Group. Cost-effectiveness of respiratory syncytial virus prevention strategies in Mozambique: a modelling study.
J Glob Health Economics and Policy. 2025;5:e2025013.

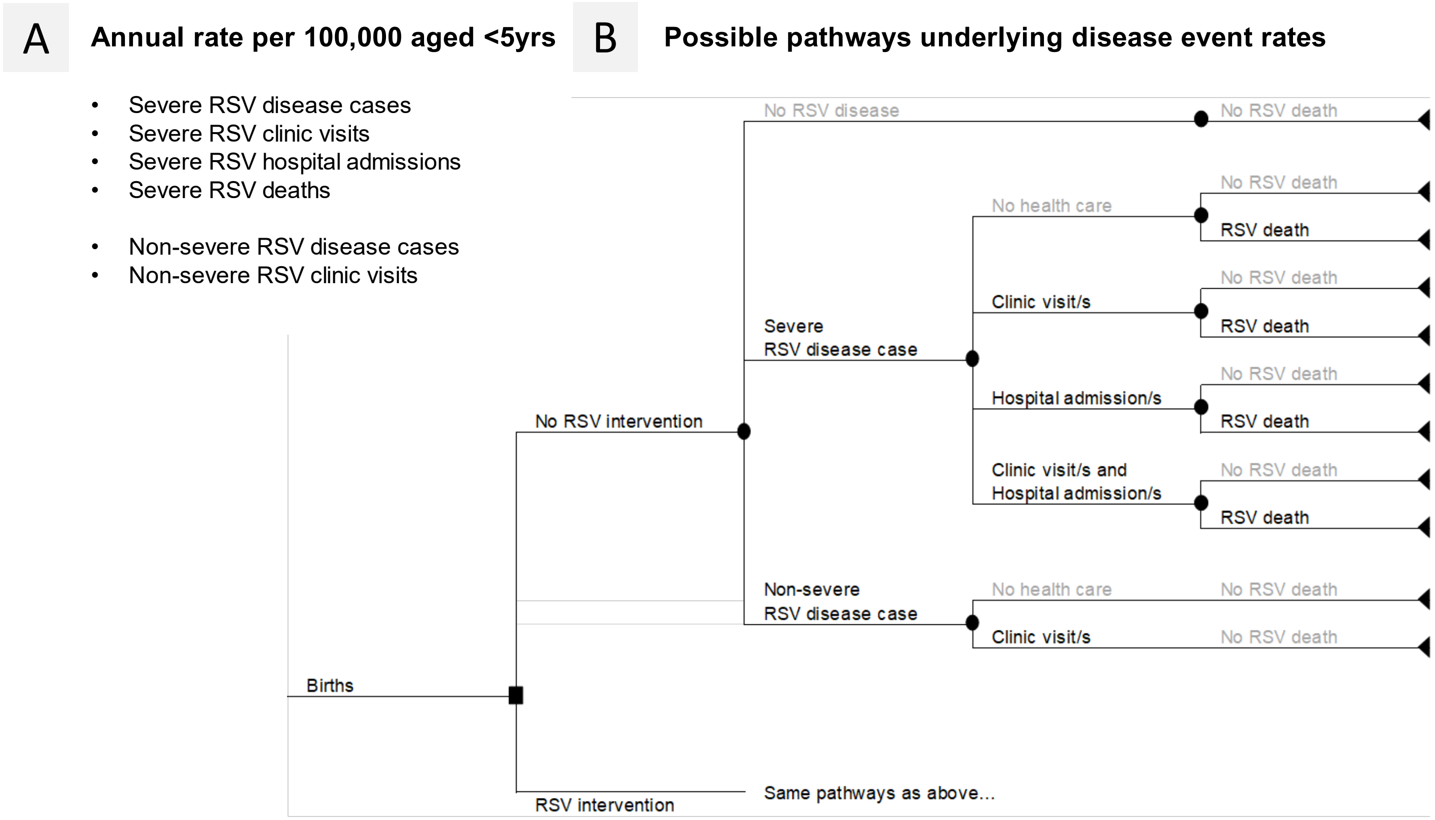

Supplementary Figure 1. Rates of RSV disease events in the recommended setup of UNIVAC, showing the possible pathways and corresponding disease event rates.

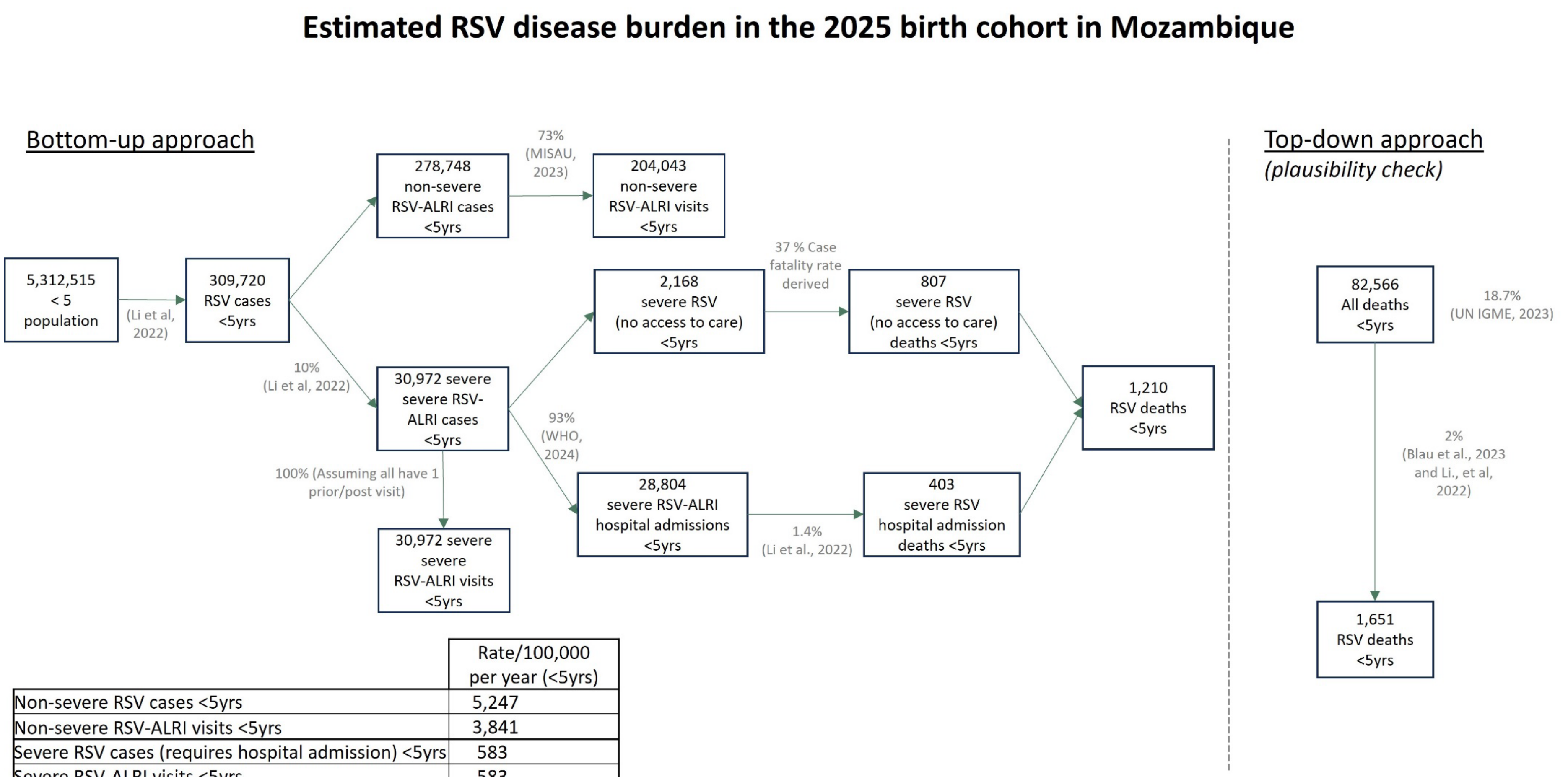

	Rate/100,000 per year (<5yrs)
Non-severe RSV cases <5yrs	5,247
Non-severe RSV-ALRI visits <5yrs	3,841
Severe RSV cases (requires hospital admission) <5yrs	583
Severe RSV-ALRI visits <5yrs	583
Severe RSV hospital admissions <5yrs	542
RSV deaths <5yrs	23

Supplementary Figure 2. Flow chart of RSV disease burden inputs in the UNIVAC model
Abbreviations: RSV – Respiratory Syncytial Virus, ALRI – Acute Lower Respiratory Infection.

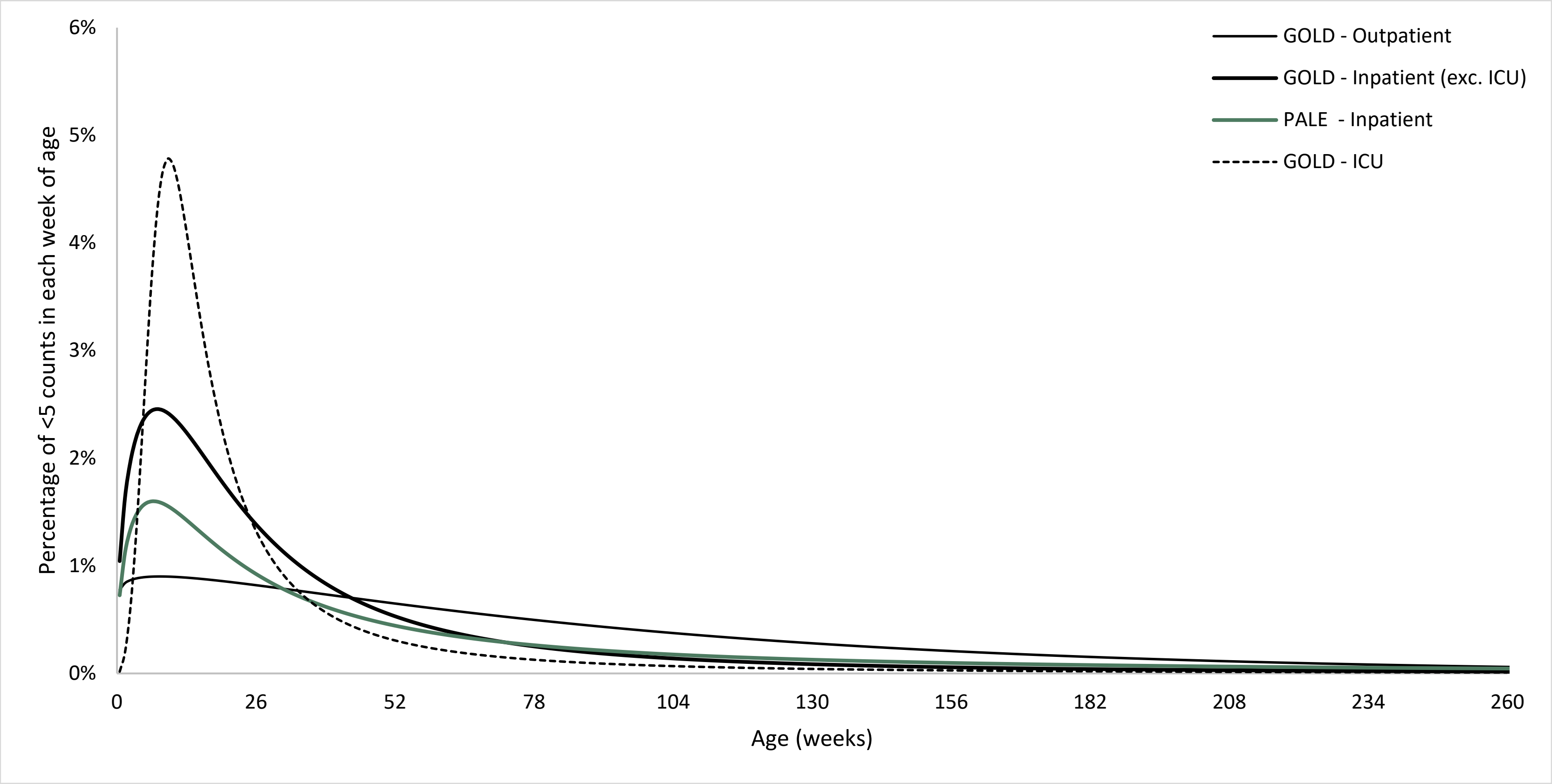

Supplementary Figure 3. Age distribution of respiratory syncytial virus presentations from studies used as input parameters for age-burden distributions in the UNIVAC model

Legend: GOLD – Global Mortality Database for Children with RSV infection[1, 2]; ICU – Intensive Care Unit; PALE – Risk factors and circulation pattern of respiratory syncytial virus in children under 2 years in Maputo[3].

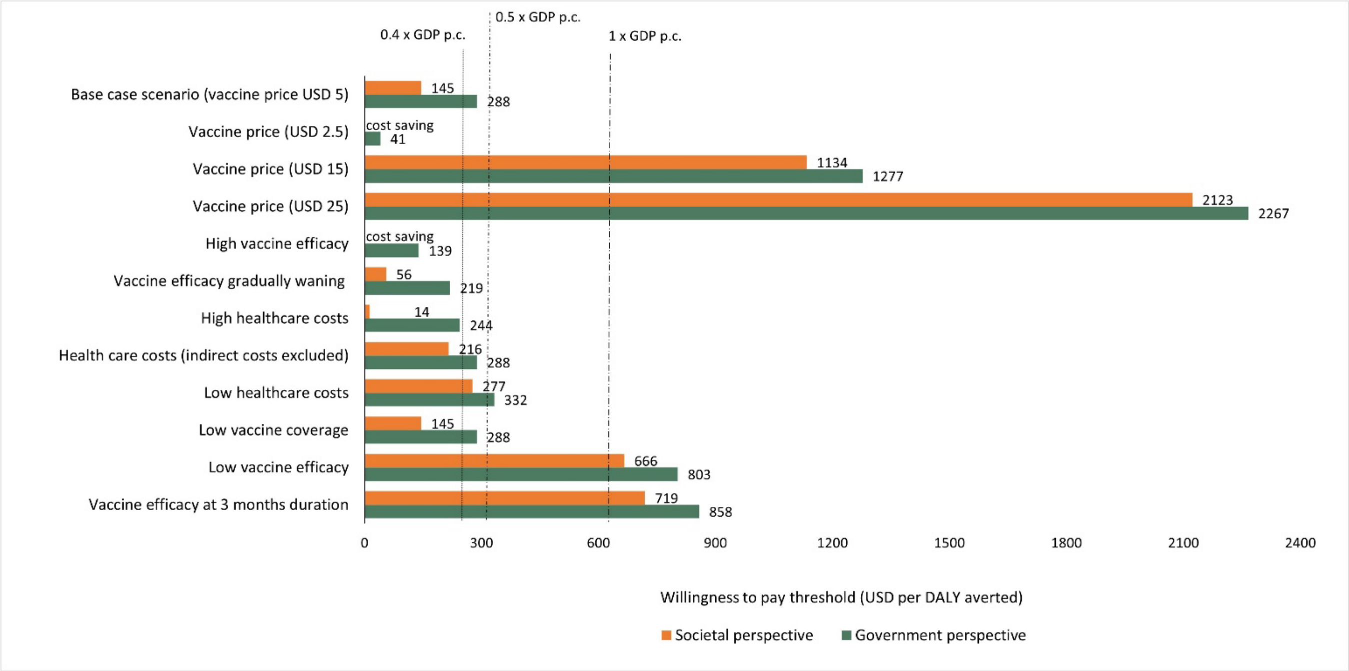

Supplementary Figure 4. Scenario analysis results showing incremental cost-effectiveness ratios (USD per disability-adjusted life year [DALY] averted) of maternal vaccine, compared to no intervention.

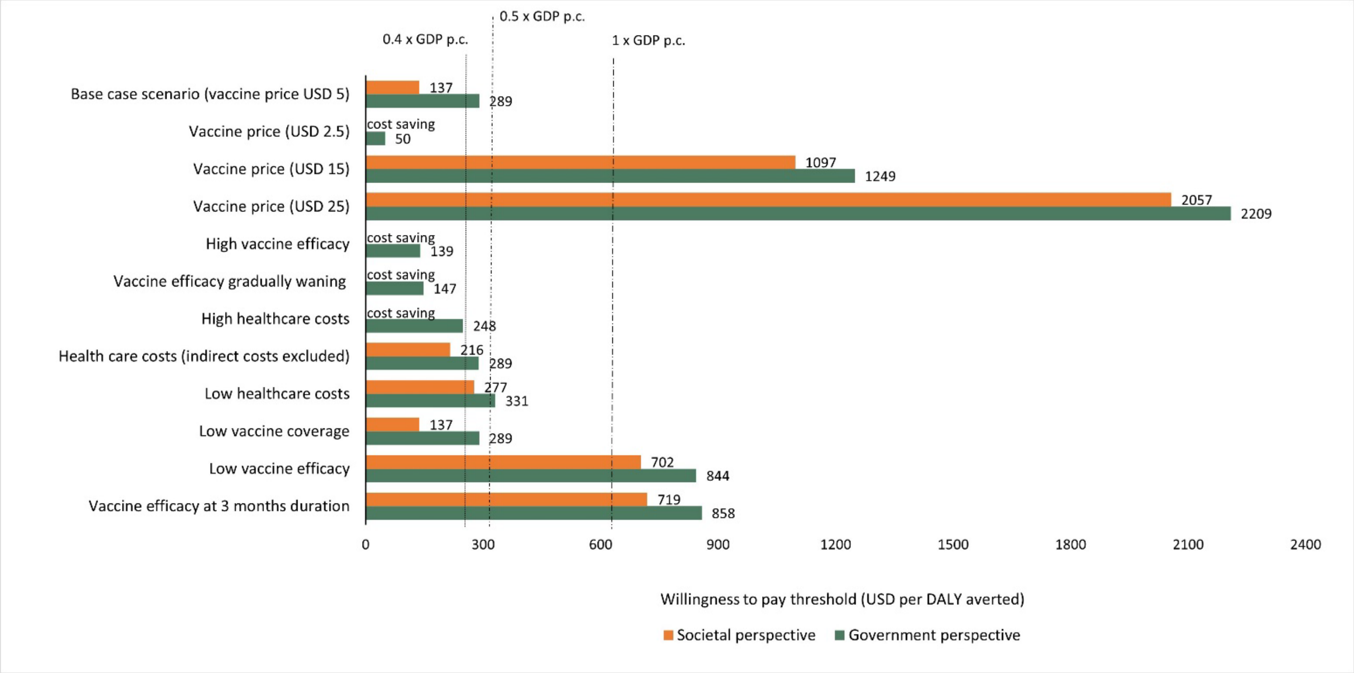

Supplementary Figure 5. Scenario analysis results, showing incremental cost-effectiveness ratios (USD per disability-adjusted life year [DALY] averted) of a long-acting infant monoclonal antibody, compared to no intervention.

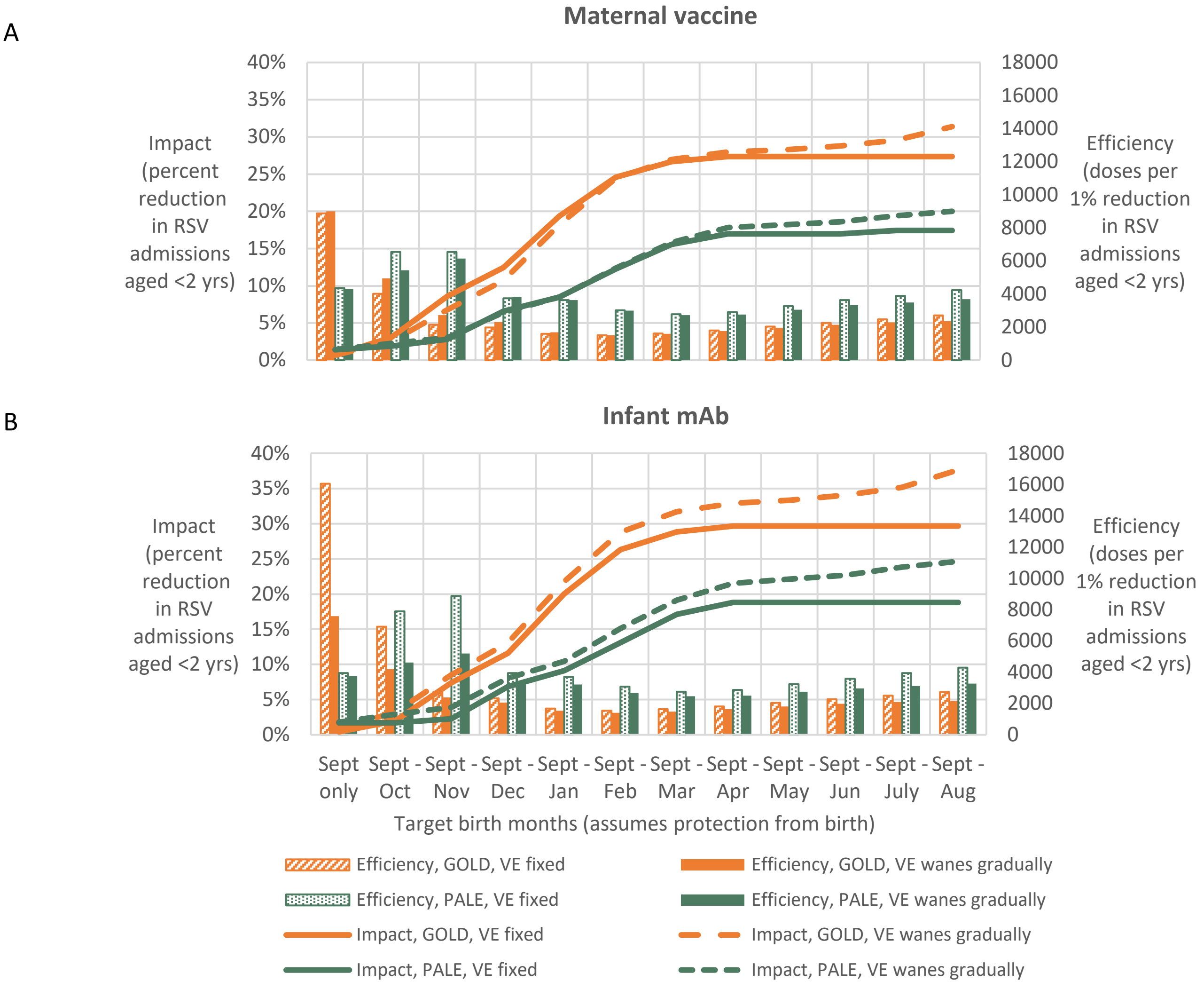

Supplementary Figure 6. Impact and efficiency of seasonal administration of respiratory syncytial virus (RSV) maternal vaccine (A) and long-acting infant monoclonal antibody (B). Legend: GOLD – Global Mortality Database for Children with RSV infection[2]; ICU – Intensive Care Unit; VE – Vaccine Efficacy; PALE – Risk factors and circulation pattern of respiratory syncytial virus in children under 2 years in Maputo[3].

References

1. RSV GOLD III - ICU Network Study Group. Respiratory syncytial virus infection among children younger than 2 years admitted to a paediatric intensive care unit with extended severe acute respiratory infection in ten Gavi-eligible countries: the RSV GOLD-ICU Network study. Lancet Glob Health. 2024.

2. Rave N, Mussá T, Nguyen A, Pecenka C, Shaaban FL, Bont LJ. Estimating the economic burden of respiratory syncytial virus infection among children <2 years old receiving care in Maputo, Mozambique. 2025.

3. Pale M, Tivane A, Gils T, Bauhofer A, Nguenha N, Machalele L, et al. Risk factors and circulation pattern of respiratory syncytial virus in children under 2 years in Maputo, Mozambique. Int Health. 2024.
